# A cross-sectional study of knowledge, attitude, and practice toward COVID-19 in solid organ transplant recipients at a transplant center in the United States

**DOI:** 10.3389/fpubh.2022.880774

**Published:** 2022-09-23

**Authors:** Lucas Wang, Mujahed Abualfoul, Hellen Oduor, Priyanka Acharya, Mingyang Cui, Anne Murray, Edward Dominguez, Mangesh Pagadala

**Affiliations:** ^1^Department of Internal Medicine, Methodist Dallas Medical Center, Dallas, TX, United States; ^2^Methodist Dallas Medical Center, Liver Institute at Methodist Dallas, Dallas, TX, United States; ^3^Clinical Research Institute, Methodist Health System, Dallas, TX, United States

**Keywords:** knowledge, attitude, practices, COVID-19, immunosuppression, liver transplant, kidney transplant, health disparities

## Abstract

**Objectives:**

Knowledge, attitude, and practices (KAP) have been widely used during times of pandemic to quantify and locate gaps of care during pandemics. Using this tool, we can identify and target populations who underwent solid organ transplant (SOT) to bolster preventative practices in these patients during COVID-19.

**Materials and methods:**

An institution-based cross-sectional study was conducted between June 1, 2020 and June 30, 2021 on patients who underwent a liver and/or kidney transplant at Methodist Dallas Medical Center in Dallas, Texas, USA. A KAP questionnaire of 26 questions about COVID-19 was designed based on the clinical and community management guidelines published by the WHO. The participant's overall KAP was categorized using Bloom's cut-off point. A KAP domain was considered sufficient if the score was between 60 and 100% and insufficient if the score was <60%. The strength of association was assessed by using odds ratio (OR); only significant independent factors in each tested area were assessed.

**Results:**

Respondents with children in the household were less likely to have sufficient practices than those who did not [OR = 0.2491, 95% Confidence Interval (0.0893–0.6120), *p* = 0.001]. We also found that sufficient levels of knowledge correlated with higher likelihood of sufficient levels of practices [OR = 4.94, 95% CI (1.646–14.2159), *p* < 0.005]. Interestingly, we found that sufficient levels of attitude did not correlate with sufficient levels of practice (*p* = 0.201).

**Conclusion:**

Our study found that knowledge and having children in the household correlated with higher levels of practice.

## Introduction

COVID-19, caused by severe acute respiratory syndrome coronavirus 2 (SARS-CoV-2), was declared to be a global pandemic by the World Health Organization (1, 2). During this time, several at risk populations were identified to have an increased risk of mortality and morbidity from COVID-19, including patients with metabolic liver disease and decompensated cirrhosis (3–6). These findings are even more pronounced in solid organ transplant (SOT) recipients (SOTRs) who are on chronic long-term immunosuppression and are vulnerable to infection. Interestingly, this data deviates from prior epidemics which haves shown that outcomes in SOTRs are comparable to the general population (7–9). While we wait for new data to accumulate for this population, the medical field can still take large strides in improving COVID-19 related outcomes in SOTRs by determining gaps of care and enforcing guidelines applicable to the general population.

Knowledge, attitudes, and practices (KAP) studies have been widely used during pandemics to quantify and locate gaps in the healthcare system (10, 11). During the initial phase of the COVID-19 pandemic, various prevention and control measures were adopted globally, including shelter-in-place policies, social distancing and telehealth (12–14). In accordance with the KAP theory, adherence to these measures is affected by the population's KAP toward the disease (15). Despite widespread concern about the impact of COVID-19 on SOTRs, COVID-19 KAP studies in the United States thus far do not include data specific to SOTRs. In this study, we report the KAP toward COVID-19 among a SOTR cohort in Dallas, Texas during the first year of the pandemic (early 2020 to mid-2021) to determine key demographics of SOTRs to target and improve areas of outreach and education.

## Materials and methods

### Study design and patient recruitment

An institution-based cross-sectional study was conducted between June 1, 2020 and June 30, 2021 on all patients who underwent a liver and/or kidney transplant at Methodist Dallas Medical Center in Dallas, Texas, USA. The sample size of this study was based on the number of transplants performed at the center during the allotted time frame. The authors factored in a 30% non-response rate. All organs were donated voluntarily with written informed consent, and this was conducted in accordance with the Declaration of Istanbul. Of note, this study was conducted at a time when the COVID-19 vaccine was not available to the general public, therefore, all participants were unvaccinated. Participants provided verbal or electronic written informed consent before completion of the KAP questionnaire. Only questionnaires returned by June 30, 2021 were considered for analysis. The study was approved by the Methodist Health System institutional review board (Protocol ID: 032.HEP.2020.D).

### Questionnaire design

A KAP questionnaire of 26 questions about COVID-19 was designed (Supplementary Document 1). The questionnaire was developed based on the clinical and community management guidelines that were published by the WHO at the time (15). The questionnaire was also piloted on 20 healthcare professionals and modified accordingly. Results from the pilot study were excluded from the final analysis. The questionnaire was administered to the patients *via* e-mail or paper during in-office visits. The first part of the questionnaire collected demographic data (e.g., age, sex, marital status, education level, number of children, zip code of residence, type of SOT, type of immunosuppressive therapy after SOT, if the patient ever tested positive for COVID-19, and if they had difficulties accessing masks). The second part of the questionnaire focused on KAP toward COVID-19. The KAP consisted of 17 questions about COVID-19. Those who answered affirmatively that they had contracted COVID-19 were asked further questions regarding their COVID-19-related symptoms and hospitalizations. The participants were also surveyed regarding immunosuppression use during the pandemic and their attitudes toward COVID-19 vaccinations.

To assess the knowledge score, seven items on the questionnaire were measured. Each correct answer was scored one point and incorrect questions were scored zero points. To assess the attitude score, six items were analyzed. Each item had three answer choices: to continue to receive optimum medical care, to reflect an indecisive attitude, or to neglect their healthcare needs. One point was assigned to those who chose to continue receiving optimal medical care and zero points were awarded for “indecisive attitude” or “neglect their healthcare needs.” To assess positive changes in practice, four items were analyzed. After the scores from all the sections were compiled, the percentage of “correct” answers out of the total was determined.

### Statistical analysis

All statistical analyses were performed using Stata 16 (StataCorp. 2019. Stata Statistical Software: Release 16. College Station, TX: StataCorp LLC). The data were analyzed using appropriate descriptive statistics and summarized by frequency and percentage. Independent variables were gender, age, marital status, number of children in household, education level, type of organ transplant, difficulty accessing a mask, tested positive for COVID-19 and triple immunosuppressant use. The percentage of correct responses was calculated by dividing the total number of correct answers by the total number of questions and multiplying that result by 100 in each tested area (i.e., knowledge, attitude, and practice). The participant's overall KAP was categorized using Bloom's cut-off point (16). For our study we only categorized into two categories: sufficient and insufficient. A KAP domain was considered sufficient if the score was between 60 and 100% and insufficient if the score was <60%. Although previous studies used Bloom's cut-off point as 80–100% being good scores, 60–79% being moderate scores, and <60% and below for poor scores; our team decided to create 2 subdivisions instead of 3. Here we chose to merge moderate and good as one and kept poor as <60%. This was done to better distribute the survey's scores into more distinct categories given the volume of questions for each KAP section. The strength of association was assessed by using odds ratio (OR) and confidence intervals (CI); only significant independent factors in each tested area were assessed. A *p* < 0.05 level was considered statistically significant.

## Results

### Demographics

Most respondents were male (58.0%), over the age of 61 (53.8%), married or in a domestic partnership (64.7%), and had children in the household (58.8%) (Table 1). In this study, 42.4% of the patients completed high school, 37.0% completed college, and 12.6% completed a post-graduate degree. In addition, most participants were liver (62.6%) or kidney transplant (34.5%) recipients. The most common reported immunosuppressants were mycophenolate (38.7%) and prednisone (49.6%). At the time of the survey 15.1% of the patients had contracted COVID-19 infection.

**Table 1:** Socio-demographic and clinical characteristics of the solid organ transplant patient study population.

**Variable, n (%)**	**Category**	**Overall** **(*N* = 238)**
Gender	Female	100 (42.0%)
	Male	138 (58.0%)
Age	<40 years	17 (7.1%)
	41–60 years	93 (39.1%)
	>61 years	128 (53.8%)
Marital status	Single	35 (14.7%)
	Married	154 (64.7%)
	Widowed	14 (5.9%)
	Divorced	35 (14.7%)
Children in household	Yes	140 (58.8%)
	No	98 (41.2%)
Education level	<High school	18 (7.6%)
	High school	101 (42.4%)
	College degree	88 (37.0%)
	Post-graduate	30 (12.6%)
Type of organ transplant	Liver	149 (62.6%)
	Kidney	82 (34.5%)
	Liver and Kidney	7 (2.9%)
Difficulty accessing a mask?	Yes	36 (15.1%)
	No	202 (84.9%)
Tested positive for COVID-19	Yes	24 (15.1%)
	No	193 (84.9%)
Immunosuppression	Steroid	118 (49.6%
	Mycophenolate	92 (38.7%)
	Tacrolimus	41 (17.2%)
	Sirolimus	14 (5.9%)
	Triple immunosuppression	76 (31.9%)

### Knowledge, attitudes, and practices of COVID-19

Most of the patients were aware that COVID-19 was caused by a type of virus (90.3%) and at the time of the survey there were no effective medications or vaccines available to cure or prevent the disease (87.0%) (Table 2). Additionally, most of the patients were aware that the virus could be spread even without signs or symptoms (95.0%) and social distancing during the peak of the pandemic was important to control the spread of the virus (86.6%). The areas with the most misconception were the clinical manifestation of COVID-19 and its mode of transmission. Less than three-quarters of the respondents knew the signs and symptoms of the infection (71.0%) and lacked adequate knowledge on the various modalities for the spread of the virus (60.9%).

**Table 2:** Knowledge responses about COVID-19 among solid organ transplant recipients (*N* = 238).

**Serial no**	**Knowledge question**	**Frequency (%)**	
		**Correct**	**Incorrect**
1	COVID-19 is caused by a novel coronavirus	90.3%	9.7%
2	COVID-19 virus spreads *via* the following method(s): respiratory droplets of infected individuals, contact with blood of infected individual, contaminated water, or all of the above.	60.9%	39.1%
3	The main clinical symptoms of COVID-19 are fever, dry, cough, shortness of breath and myalgia, chest pain or shortness of breath, only fever, abdominal pain, and diarrhea, or all of the above.	71.0%	29.0%
4	Although COVID-19 can affect everybody, the populations that are mostly likely to develop severe symptoms are elderly people, patients suffering from ailments such as heart disease, diabetes, blood pressure and immunocompromised (low immunity) individuals	95.0%	5.0%
5	Currently there is no available cure for COVID-19	87.0%	13.0%
6	COVID-19 can also be spread by individuals who do not have symptoms	95.0%	5.0%
7	Social distancing will help reduce and eliminate COVID-19 disease?	86.6%	13.4%

Almost three-fourths (73.1%) of the SOTRs had a positive attitude toward vaccination and if made available were open to receiving immunization (Table 3). Additionally, most were willing to encourage friends or family members to consider vaccination (77.3%). Half (53.8%) of the patients surveyed stated that they would have been more inclined to decline a SOT during the COVID-19 pandemic if they were still on the transplant list. Less than half (45.4%) of the respondents stated that they would avoid seeking routine health care visits, if possible, until the pandemic had resolved.

**Table 3:** Attitude responses toward COVID-19 among solid organ transplant recipients (*N* = 238).

**Serial no**	**Attitude question**	**Frequency (%)**	
		**Correct**	**Incorrect**
A1	If you develop fever or cough with sore throat, are you more likely to self-quarantine for 14 days and only get tested if fever or symptoms worsen, get tested for COVID-19 immediately, or continue normal routine.	45.8%	54.2%
A2	If you were to receive an organ for liver or kidney transplant during this COVID 19-9 epidemic (assuming you were still on waitlist) you would accept the organ, decline the transplant, accept only if donor was checked for COVID-19?	53.8%	47.2%
A3	In your opinion, what is the best way to seek health care during the COVID-19 pandemic?	45.4%	54.6%
A4	Do you believe that COVID-19 can be prevented by vaccination?	74.8%	25.2%
A5	If a vaccination of COVID-19 is available, would you like to be vaccinated?	73.1%	26.9%
A6	Will you advice your relatives and family to obtain immunization of COVID-19 vaccine	77.3%	22.7%

In response to practices toward COVID-19 prevention, most of the respondents reported that the COVID-19 pandemic had increased their overall awareness and knowledge of maintaining hygiene, including taking sanitary precautions in public places (83.2%) and hand washing (85.3%) (Table 4). Despite improved awareness, less than half the patients were willing to wear a mask in appropriate situations (42.9%).

**Table 4:** Practice responses toward COVID-19 among solid organ transplant recipients (*N* = 238).

**Serial no**	**Practice question**	**Frequency (%)**	
		**Correct**	**Incorrect**
P1	As a transplant recipient, has the COVID-19 pandemic increased your awareness and knowledge of maintaining hygiene, including taking precautions in public places to avoid contracting other infections in the future?	83.2%	16.8%
P2	Has the COVID-19 pandemic increased your practice of hand sanitation?	85.3%	14.7%
P3	What do you use to wash/clean your hands?	99.6%	0.04%
P4	When are you likely to wear a mask?	42.9%	57.1%

We also see that those with children in the household were less likely to have sufficient practices than those who did not (OR = 0.2491, 95% CI (0.0893–0.6120), *p* = 0.001) (Table 5). We also found that sufficient levels of knowledge correlated with higher likelihood of sufficient levels of practices [OR = 4.94, 95% CI (1.646–14.2159), *p* < 0.005]. However, there was not a significant correlation between sufficient levels of attitude and sufficient levels of practice (*p* = 0.201) (Table 5).

**Table 5:** Sociodemographic determinants of sufficient practice scores.

**Variables [N (%)]**	**Practice**		***p*-Value** **odds ratio** **95% confidence interval**
	**Insufficient *N* = 40**	**Sufficient *N* = 198**	
**Knowledge** Insufficient Sufficient	9 (22.50) 31 (77.50)	11 (5.56) 187 (94.44)	**< 0.005** **OR** **=** **4.9355** **95% CI** **=** **[1.646, 14.2159]**
**Attitude** Insufficient Sufficient	18 (45.00) 22 (55.00)	68 (34.34) 130 (65.66)	0.201
**Gender** Male Female	24 (60.00) 16 (40.00)	114 (57.58) 84 (42.42)	0.777
**Age** <40 41–60 years >60 years	2 (5.00) 22 (55.00) 16 (40.00)	15 (7.58) 71 (35.86) 112 (56.57)	0.083
**Marital status** Single Married Widowed Divorced	6 (15.00) 26 (65.00) 1 (2.50) 7 (17.50)	29 (14.65) 128 (64.65) 13 (6.57) 28 (14.14)	0.816
**Children in household** No Yes	7 (17.50) 33 (82.50)	91 (45.96) 107 (54.04)	**0.001** **OR** **=** **0.2491** **95% CI** **=** **[0.0893, 0.6120]**
**Education level** <High school High school College degree Post-graduate	1 (2.56) 17 (43.59) 15 (38.46) 6 (15.38)	17 (8.59) 84 (42.42) 73 (36.87) 24 (12.12)	0.601
**Type of organ transplant** Liver Kidney Liver and Kidney	20 (50.00) 17 (42.50) 3 (7.50)	129 (65.15) 65 (32.83) 4 (2.02)	0.051
**Difficulty accessing a mask**. No Yes	37 (92.5) 3 (7.5)	165 (83.33) 33 (16.67)	0.104
**Tested positive for COVID-19** No Yes	30 (83.33) 6 (16.67)	163 (90.06) 18 (9.94)	0.240
**Triple immunosuppressant** No Yes	26 (65.00) 14 (35.00)	136 (68.69) 62 (31.31)	0.648

Bolded values represent statistically significant comparison (*p* < 0.05).

## Discussion

To our knowledge, this was the first study to assess the KAP of SOTRs in the United States during the early stage of the pandemic in 2020. The United States imposed a stringent lockdown across the country for several months to control the spread of COVID-19. Though there has been a vaccine rollout, the spread of new variants of SARS-CoV-2 continues to be rampant in the United States and in many countries around the world. Therefore, despite vaccination there continues to be an immediate need for effective prevention at various levels of public health. Adequate prevention measures could be successful by enhancing the KAP of the population.

The COVID-19 pandemic has increased the risk of mortality and morbidity in those with chronic illnesses including those with liver disease and cirrhosis (3–6). SOTRs have been shown to have higher rates of infection and complications from COVID-19 due to use of immunosuppressive medications. They also have more comorbidities than the general population. The pandemic has increased anxiety levels among SOTRs, leading to modifications to their daily lifestyle (17, 18).

The results of our study showed participants achieved a mean score of 83.3% in the knowledge portion of the questionnaire. These results are comparable to those in studies done in both the general population and high-risk populations (19, 20). Therefore, adequate knowledge could have led to better social distancing practices and sanitary precautions leading to a lower incidence of COVID-19 among SOTR. These findings highlight the need to continue to encourage and emphasize maintaining social distancing as a means of preventing the spread of COVID-19. We also found that only a quarter of the respondents shared a negative attitude toward COVID-19 vaccination including not wanting their relatives to be vaccinated. Interestingly, the mean score in the attitude portion of the questionnaire (61.7%) was relatively lower than those in studies from other countries (21). Hesitancy rates for vaccination in our SOTR cohort were comparable to the national trend suggesting that SOTR attitudes toward vaccination were similar to the general US population (22). To achieve higher vaccination rates, it is important to implement adequate policies and use various media platforms to address the safety aspects and existing misconceptions of the COVID-19 vaccines. During the pandemic, routine office consultation visits were canceled or deferred by patients due to increased fear of contracting an infection at a health care facility when compared to staying at home. In our study, 54.6% of the SOTRs wanted to avoid or minimize regular office visits. As the duration of the pandemic progresses, it is more likely that SOTRs might postpone health care visits. One way to deliver better health care during the COVID-19 pandemic is to bolster the practice of medical care *via* telehealth. Similarly, there was an overall decline in all SOT especially among kidney and living donors during 2020 compared to 2019 (7). Almost 47.2% stated that they would decline an offer for a transplant during the pandemic. It is possible that once again the fear of contracting an infection from a healthcare setting and the increase in the availability of deceased donors from deaths related to COVID-19 could have resulted in these results. To ameliorate this concern, the United Network for Organ Sharing has made it mandatory to test all donors and recipients for COVID-19 and make this information available to patients on the waiting list.

As expected, SOTRs with sufficient knowledge scores reported to adhere to higher levels of practices compared to those with insufficient scores. This finding underlines the importance of robust communication strategies in vulnerable populations. Another factor that correlated to higher levels of practice were if patients had children living in the household at the time of survey. A similar study conducted by Alremeithi et al. also reflects this finding. A possible explanation for this is that parents would want to pursue and project a mindset for their children to emulate to prevent contracting COVID-19. Furthermore, it was interesting to see that mask accessibility did not have a significant impact on reporting good attitude or practices around COVID-19. Given the time when this survey was conducted, this could suggest that in the initial stages of the pandemic SOTRs may have faced geographic or financial obstacles keeping them from maintaining awareness and good practices regarding COVID-19.

### Study limitations

Some limitations should be considered when interpreting the findings of our study. The data were from self-reported information, increasing the chance of reporter bias. The use of a small sample size and the cross-sectional nature of the study does not allow for determination of any cause-and-effect relationships. Given that all patients surveyed were from Methodist Dallas Medical Center, the results of the study may not reflect the KAPs of SOTRs worldwide. Additionally, the survey responses were received over different time points in the pandemic. Those who responded later could have had a different perspective on the pandemic compared to those who responded earlier due to better dissemination of knowledge and information.

## Conclusion

To our knowledge, this was the first KAP study conducted among SOTRs in the United States. The immunosuppressed state of the cohort makes them vulnerable to infection with higher morbidity compared to the general population. We found that having sufficient knowledge about COVID-19 does correlate with maintaining higher standards of practices in preventing infection. However, in this study we also found that a significant amount of SOTRs. This may represent a key point in the healthcare battle against COVID-19 as it insinuates that improving education of the disease may lead to increased prevention of contraction from a mass population perspective. Overall, this study demonstrates that a more comprehensive understanding of COVID-19 correlated with an increased adherence to practices to prevent its spread. Based on these results, we strongly recommend bolstering avenues for education, particularly amongst patient populations at most risk for worse COVID-19 outcomes. In the end, we believe that a better-informed population will lead to one that adheres to COVID-19 preventative practices.

## Data availability statement

The raw data supporting the conclusions of this article will be made available by the authors, without undue reservation.

## Ethics statement

The studies involving human participants were reviewed and approved by Methodist Health System Institutional Review Board (Protocol 032.HEP.2020.D). The patients/participants provided their written informed consent to participate in this study.

## Author contributions

LW: organized and participated in data gathering and primary manuscript author. MA: participated in data gathering. HO: secondary manuscript author. PA: primary statistician. MC: secondary statistician. ED: reviewer and editor. MP: Principal Investigator and editor. All authors listed have made a substantial, direct, and intellectual contribution to the work and approved it for publication.

## Conflict of interest

Authors PA, MC, and AM were employed by Methodist Health System. The remaining authors declare that the research was conducted in the absence of any commercial or financial relationships that could be construed as a potential conflict of interest.

## Publisher's note

All claims expressed in this article are solely those of the authors and do not necessarily represent those of their affiliated organizations, or those of the publisher, the editors and the reviewers. Any product that may be evaluated in this article, or claim that may be made by its manufacturer, is not guaranteed or endorsed by the publisher.
