# Supplementary material for: A cross-sectional study of knowledge, attitude, and practice toward COVID-19 in solid organ transplant recipients at a transplant center in the United States
Source: Front Public Health. 2022 Sep 23;10:880774. doi: 10.3389/fpubh.2022.880774 (PMC9539443; doi:10.3389/fpubh.2022.880774)
Supplement: Supplementary file 1 [file Data_Sheet_1.pdf]

COVID KAP Liver and Kidney Transplant Patient Survey

1) First Name:

2) Last Name:

3) Email Address:

4) Phone Number:

5) Date of Birth:

6) Gender:

7) Marital Status (Single = 1, Married or Domestic Partnership = 2, Widowed = 3, Separated = 4, Divorced = 5):

8) Number of Children in Household:

9) Education Level (Less than High School = 1, High School Diploma = 2, College Degree = 3, Postgraduate Degree = 4, Divorced = 5):

10) Highest Education Level of Mother (Less than High School = 1, High School Diploma = 2, College Degree = 3, Postgraduate Degree = 4, Divorced = 5):

11) Highest Education Level of Father (Less than High School = 1, High School Diploma = 2, College Degree = 3, Postgraduate Degree = 4, Divorced = 5):

12) City of Residence:

13) Zip code:

14) Language(s) spoken:

15) Have you tested positive for COVID-19?

16) Were you admitted to the hospital due to COVID-19?

17) If yes, on what dates were you admitted?

18) To which hospital were you admitted?

Knowledge

Please select all correct answers for full credit on questions.

19) COVID-19 is caused by the novel coronavirus (**True = 1**, False = 2):

20) COVID-19 virus spreads via the following methods **(A) Respiratory droplets of infected individuals = 1**, B) Contact with blood of infected individual, C) Contaminated water, D) All of the above):

21) The main clinical symptoms of COVID-19 are **(A) Fever, dry cough, shortness of breath and myalgia (body ache) = 1, B) Chest pain and shortness of breath,** C) Only fever, **D) Abdominal pain and diarrhea):**

22) Although COVID -19 can affect everybody, the populations that are mostly likely to develop severe symptoms are elderly people, patients suffering from ailments such as heart disease, diabetes, blood pressure and immunocompromised (low immunity) individuals (**True = 1**, False = 2):

23) Currently there is no available cure for COVID-19 (**True = 1**, False = 2):

24) Patients who undergo transplant and are on immunosuppressive medication are at higher risk for contracting COVID-19 (**True = 1**, False = 2):

25) COVID -19 can also be spread by individuals who do not have symptoms (True = 1, False = 2):

26) Social distancing will help reduce and eliminate COVID-19 disease (**Yes = 1**, No = 2, Do not Know = 3)?

Attitudes

Please select the choice you most identify with. Each question may have multiple answers that will result in full credit.

27) If you develop fever or cough with sore throat are you more likely to (A) Self-quarantine for 14 days and only get tested if fever or symptoms worsen = 1, **B) Self-quarantine and get tested for COVID-19 immediately = 2**, C) No self-quarantine but will get tested for COVID-19, D) No self-quarantine and will not get tested for COVID-19)?

28) If you were to receive an organ for liver or kidney transplant during this COVID 19 epidemic (assuming you were still on waitlist) you would **(A) Accept the organ = 1**, B) Decline a transplant = 2, **C) Accept only if donor was checked for COVID-19 = 3)**?

29) In your opinion, what is the best way to seek health care during the COVID -19 pandemic? **(A) I do not mind visiting the doctor's office = 1, B) Avoid the doctor's**

office/clinic and do a health visit over the phone = 2, C) Avoid health care facilities at all costs = 3).

30) Do you believe that COVID-19 can be prevented by vaccination (**Yes = 1**, No = 2)?

31) If a vaccination of COVID-19 is available, would you like to be vaccinated (**Yes = 1**, No = 2)?
32) Will you advice your relatives and family to obtain immunization of COVID-19 vaccine (**Yes = 1**, No = 2)?

Practices

Please select the choice you most identify with. Each question may have multiple answers that will result in full credit.

33) As a transplant recipient, has the COVID -19 pandemic increased your awareness and knowledge of maintaining hygiene, including taking precautions in public places to avoid contracting other infections in the future (**Yes = 1**, No = 2, Not any different from before = 3)?

34) Has the COVID -19 pandemic increased your practice of hand sanitation (**Yes = 1**, No = 2)?

35) What do you use to wash/clean your hands **(A) An alcohol-based hand sanitizer like PURELL = 1**, **B) Disinfectant soap in a bottle = 2**, **C) Soap and Water = 3, D) All of the above = 4**, E) None of the above = 5)?

36) When are you likely to wear a mask (A) Only when you visit a public place like a grocery store etc. = 1, B) Only when you visit a health care facility (clinic/hospital) = 2, C) When you come in contact with any individual = 3, D) All of the time, even at home = 4, **E) Only A, B, C = 5, F)** I rarely use a face mask = 6)?

Miscellaneous

37) What kind of mask do you prefer to use (A) Cloth mask = 1, B) Surgical Mask = 2, C) N95 mask = 3)?

38) Do you have difficulties accessing the type of mask you most like to wear (Yes = 1, No = 2)?

39) In your opinion, what is the one thing that has been the most important in protecting you from COVID-19?

40) Have you heard about a potential vaccination for COVID-19 (Yes = 1, No = 2)?

41) If a vaccination of COVID-19 is available would you like to be vaccinated (Yes = 1, No = 2, Not Sure = 3)?

42) Will you advice your relatives and family to obtain immunization of COVID-19 vaccine (Yes = 1, No = 2)?

43) Did you visit a healthcare facility such as an ER, hospital, or physician's office within 4 weeks prior to contracting COVID-19 infection (Yes = 1, No = 2)?

44) If yes, did you visit an ER, a hospital, or a physician's office (A) ER, B) ER and hospital C) Physician's office)?

45) Did any of your family members living with you contract COVID-19 prior to your infection (Yes = 1, No = 2)?

46) Did you meet any family members or friends who recently had COVID-19 infection (Yes = 1, No = 2)?

47) Did you have a family gathering 4 weeks prior to contracting the COVID-19 infection (Yes = 1, No = 2)?

48) Do your immediate family wear mask regularly when you venture outside of your home (Yes = 1, No = 2, Some of the time = 3)?

49) In your opinion, how did you contract the COVID-19 infection?

50) What were your symptoms for COVID-19 (Sinus pressure = 1, Headache = 2, Nasal Stuffiness = 3, Nasal Discharge = 4, Loss of smell = 5, Sore throat = 6, Fever = 7, SoB = 8)
